# Differential associations of occupational stress dimensions with job burnout subscales among nurses

**DOI:** 10.3389/fpubh.2026.1774820

**Published:** 2026-03-25

**Authors:** Wenyu Zhang, Haiyang Li, Wenyan Shi, Cunduo Jin

**Affiliations:** 1 School of Nursing, Capital Medical University, Beijing, China; 2 General Practice Department, The Seventh Medical Center of Chinese PLA General Hospital, Beijing, China; 3 Cadre Diagnosis and Treatment Department, The Seventh Medical Center of Chinese PLA General Hospital, Beijing, China; 4 Department of Nursing, The Seventh Medical Center of Chinese PLA General Hospital, Beijing, China

**Keywords:** heterogeneous associations, interpersonal relationships, job burnout, nurses, occupational stress

## Abstract

**Background:**

The differential associations through which specific dimensions of occupational stress contribute to distinct components of job burnout among nurses remain insufficiently elucidated, particularly in the post-pandemic context. This study aimed to assess the status of occupational stress and burnout and, crucially, to investigate the specific associations between stress dimensions and burnout components among clinical nurses.

**Methods:**

A cross-sectional study was conducted and 3,654 registered nurses who completed an anonymous online survey. Validated instruments were utilized to assess job burnout and nursing occupational stress across multiple domains. Multivariable logistic regression analyses were performed to identify occupational stress factors independently associated with each dimension of job burnout.

**Results:**

Nurses reported mild-to-moderate overall occupational stress (mean total score = 64.19 ± 21.20; mean item score = 1.83 ± 0.61). However, burnout levels were notable, with scores of 21.33 ± 5.87 for emotional exhaustion (EE), 10.06 ± 3.20 for depersonalization (DP), and 23.13 ± 4.23 for personal accomplishment (PA). All five stress dimensions showed significant positive correlations with EE (*r* = 0.436–0.542) and DP (*r* = 0.345–0.489), and weak negative correlations with PA (*r* = −0.122 to −0.150). Regression analyses revealed distinct patterns of association: EE was independently associated with all five stress dimensions, most strongly with “nursing management and interpersonal relationships” (NMIR) (OR = 3.738, 95% CI: 2.294–6.089, *p* < 0.001) and “workload and time allocation” (WTA) (OR = 2.801, 95% CI: 2.205–3.559, *p* < 0.001). DP was specifically associated with “patient nursing care” (OR = 2.401, 95% CI: 1.564–3.688, *p* < 0.001), NMIR (OR = 2.036, 95% CI: 1.139–3.639, *p* = 0.016), and “work environment and resources” (OR = 1.486, 95% CI: 1.075–2.054, *p* = 0.017). For low PA, only NMIR (OR = 3.415, 95% CI: 1.772–6.583, *p* < 0.001) and WTA (OR = 0.556, 95% CI: 0.347–0.890, *p* = 0.014) were significant independent factors, with NMIR stress being the strongest correlate.

**Conclusion:**

This study identifies a disconnect between moderate stress perception and significant burnout, highlighting heterogeneous associations linking specific stress dimensions to different burnout components. It underscores stress related to management and interpersonal relationships as a central, pervasive factor associated with burnout. Interventions targeting organizational leadership and the relational climate are essential for mitigating burnout and fostering nurse well-being.

## Introduction

The well-being of the nursing workforce is of paramount importance to healthcare quality, patient safety, and institutional efficiency, given their role as the primary providers of continuous, direct patient care (1, 2). However, this vital profession is confronting a worldwide burnout crisis, a widespread occupational health concern with significant adverse implications (3). Job burnout is defined as a psychological syndrome stemming from chronic and unmanaged workplace stress. It is characterized by three dimensions: feelings of energy depletion or exhaustion (emotional exhaustion), increased mental distance from, or feelings of negativism or cynicism related to one’s job (depersonalization), and a sense of ineffectiveness or lack of accomplishment (low personal accomplishment) (4). The nursing workforce is particularly susceptible to this syndrome due to the inherent nature of the profession, which involves high workloads, substantial emotional demands, uncertainty, and irregular shift work (5). Extensive research indicates that chronic stress in healthcare settings not only precipitates burnout but also adversely affects the overall well-being of healthcare workers (6, 7).

In China, with the deepening implementation of the “Healthy China 2030” strategy and ongoing healthcare reforms have amplified societal demand and expectations for nursing services. Consequently, the professional requirements and workload faced by nurses have intensified (8). Occupational stress, encompassing various stressors within the nursing environment, can disrupt physical and mental equilibrium, undermine organizational development, and impair individual health (9, 10). Common stressors include nursing risk events, low work adaptability, and anxiety regarding potential errors. Furthermore, prolonged exposure to a constellation of occupational hazards, including high patient acuity, emotional labor, administrative burdens, and shift work, a well-established precursor to the onset and progression of job burnout. Previous literatures suggests that job stress may also indirectly influenced burnout by perceived social support and psychological capital. Specifically, higher levels of job stress are associated with lower resilience and increased compassion fatigue among nurses (11, 12).

While existing studies have highlighted the prevalence of occupational stress and burnout among Chinese nurses, a critical gap remains in the literature. Most research has treated occupational stress or job burnout as monolithic constructs or unidimensional constructs, failing to adequately elucidate how specific dimensions of occupational stress differentially influence the three core dimensions of burnout. To address this gap, this study aims to assess the status of job burnout and occupational stress among nurses in Beijing. By employing a multidimensional analytical approach, we seek to delineate the specific associations between distinct occupational stress dimensions and the individual components of job burnout. The findings are intended to provide an empirical basis for healthcare institutions to develop targeted intervention strategies, thereby offering workforce stability and enhancing the resilience of the healthcare system.

## Methods

### Study design and setting

This cross-sectional study was conducted from January 2023 to September 2024 among registered nurses in Beijing, China. It serves as part of a broader longitudinal investigation into the professional development of regional nursing cohorts. The study protocol, including the questionnaire structure and methodology, underwent three rounds of rigorous expert review by specialists from the Chinese PLA General Hospital, Peking Union Medical College, and Capital Medical University to ensure content validity.

Eligible participants were full-time registered nurses employed in clinical settings within Beijing. Inclusion criteria were defined as follows: (1) possession of a valid Chinese nursing license; (2) a minimum of 2 years of full-time clinical nursing experience; and (3) prior involvement in the management of acute public health challenges. Exclusion criteria comprised: (1) refusal to participate; (2) absence of valid licensure; (3) a diagnosed psychiatric disorder or severe chronic condition potentially impairing work capacity (e.g., severe depression or uncontrolled cardiovascular disease); and (4) holding non-clinical positions (e.g., exclusively administrative roles). This study was conducted in accordance with the Declaration of Helsinki and was approved by the Medical Ethics Committee of Capital Medical University (Approval No. Z2023SY051).

### Sampling strategy and data collection

To ensure a representative sample of the nursing workforce in Beijing, a stratified cluster sampling design was implemented. First, hospitals in Beijing were stratified based on three key criteria: hospital grade (tertiary, secondary, or primary), geographic location (urban, suburban, or exurban), and institutional ownership (public or private). Within each stratum, purposive sampling was applied to select hospitals deemed representative of the stratum characteristics, yielding a total of 14 participating hospitals. Within each selected hospital, a census sampling method was employed. Rather than selecting a subset of nurses, all registered nurses listed in the personnel registries of the participating hospitals who met the eligibility criteria were invited to participate. This approach eliminated selection bias at the individual level within the participating units.

The minimum required sample size was calculated using the formula *N* = (*Uασ*/*δ*)^2^. Based on a standard deviation (σ) of 1.15, a significance level (α) of 0.05 (corresponding to Zα = 1.96 for a two-tailed test), and a precision level (δ) of 0.066, the initial minimum sample size was determined to be 1,166 (1). Accounting for a potential 10% attrition rate, the recruitment target was adjusted to 1,295. Data collection was conducted using the SO JUMP platform^1^. An anonymous survey link, configured to allow only one submission per respondent, was distributed to all eligible nurses via institutional communication channels and professional networks. Prior to participation, all individuals received information about the study’s purpose and procedures and provided electronic informed consent, which they were required to confirm before starting the survey. Participation was entirely voluntary, and respondents could withdraw at any time without providing a reason.

### Questionnaire development

The survey comprised three sections. The part one aimed to gather sociodemographic characteristics, including gender, age, marital status, education, income per month Renminbi (RMB), positional titles, labor relationship.

The part two was used to assess occupational stress using the scale developed by Xiaomei and Yanjun (13), which was made and revised based on the nurse occupational stress scale from the United States and the United Kingdom, according to China’s national conditions, and is currently the most used scale for investigation on nurse occupational stress in China. It comprises 35 items across five dimensions: nursing profession and work (7 items), workload and time allocation (5 items), work environment and resources (3 items), patient nursing care (11 items), and nursing management and interpersonal relationships (9 items). Items are rated on a 4-point Likert scale (1 = “mild” to 4 = “severe”). The total score reflects the overall stress level. Based on the average item scores, stress levels were categorized as follows: 1.00–2.00 indicated mild stress, 2.01–3.00 indicated moderate stress, and 3.01–4.00 indicated severe stress. The scale demonstrated excellent internal consistency (Cronbach’s alpha = 0.98) (13, 14).

The part three was provided to investigate the job burnout among nurses using the Maslach Burnout Inventory (MBI) provided by Alacacioglu et al. (15), which contain 22-items and had three subscales: emotional exhaustion (EE, 9 items), depersonalization (DP, 5 items) and personal accomplishment (PA, 8 items). Items were rated on a 5-point frequency scale (0 = “never” to 4 = “every day”). The final score of each subscale was calculated by summing the scores of each item. Subscale scores were summed, with burnout severity defined by established cut-off points: high EE (≥27), high DP (≥10), and low PA (≤33) (15).

### Data management and quality control

Given the use of an online survey platform, procedural controls were implemented to ensure data integrity. The survey was configured with mandatory fields to prevent missing data at the source. Following data collection, raw data were exported directly from the SO JUMP platform into Microsoft Excel, eliminating manual entry errors. A rigorous data cleaning protocol was applied to identify invalid submissions based on two criteria: (1) a completion time of less than 120 s, suggesting insufficient attention to item content; and (2) discernible response patterns (e.g., straight-lining or identical answers for all questions). These invalid responses were excluded from the final dataset prior to statistical analysis.

To mitigate common method bias (CMB), several procedural remedies were employed during the survey design: (1) ensuring respondent anonymity to reduce social desirability bias; (2) incorporating both positively and negatively worded items (reverse scoring); and (3) structurally separating predictor and outcome variables within the questionnaire. Additionally, Harman’s single-factor test was conducted via exploratory factor analysis, revealing that the first factor accounted for 26.2% of the total variance (well below the critical threshold of 40%). These procedural and statistical measures confirmed that CMB did not substantially affect the study results.

### Statistical analysis

Data were analyzed using SPSS 23.0 (IBM, Armonk, NY, United States). Demographic characteristics were summarized using descriptive statistics. To identify independent influence factors for each burnout dimension, multivariate binary logistic regression analyses were conducted. The “Enter” method was employed to simultaneously include all five occupational stress dimensions as independent variables and all sociodemographic characteristics (gender, age, marital status, education, income, professional title, and labor relationship) as covariates. However, as these demographic variables did not show statistically significant associations with the outcome variables in the adjusted models, the final presentation focuses on the significant associations between occupational stress dimensions and burnout components.

The burnout subscales (EE, DP, and PA) were dichotomized were dichotomized based on the aforementioned cut-off points (15). Multicollinearity was assessed using the variance inflation factor (VIF < 5). Model fit was evaluated using the Hosmer-Lemeshow test (*p* > 0.05). To verify robustness and address information loss from dichotomization, sensitivity analyses were performed using multiple linear regression with continuous burnout scores. A two-tailed *p*-value < 0.05 was considered statistically significant.

## Results

### Sample characteristics

A total of 3,900 eligible nurses from the 14 participating hospitals were invited to participate in this study. Of these, 3,654 provided complete and valid responses, yielding an effective response rate of 93.7%. The sample was predominantly female (95.2%), under 40 years of age (84.9%), and married (60.6%). In terms of professional background, 76.7% held a bachelor’s degree, and 72.5% reported a monthly income exceeding 8,000 RMB. Senior professional titles (e.g., vice professor/chief nurse) were held by only 2.8% of participants. The majority (64.0%) were employed on a contract basis (Table 1).

**Table 1:** Demographic characteristics of among nurses in Beijing (*N* = 3,654).

Variables	Frequency (%)
Gender	
Male	172 (4.7)
Female	3,482 (95.2)
Age	
≤40	3,103 (84.9)
41–55	541 (14.8)
≥56	10 (0.3)
Marital status	
Unmarried	1,383 (37.8)
Married	2,215 (60.6)
Divorced	46 (1.3)
Others	10 (0.3)
Education	
Secondary vocational education	39 (1.1)
Higher vocational education	763 (20.9)
Bachelor’s degree	2,804 (76.7)
Master’s degree	48 (1.3)
Income per month, RMB	
≤3,500	136 (3.7)
3,501–5,000	194 (5.3)
5,001–8,000	675 (18.5)
8,001–10,000	1,513 (41.4)
≥10,001	1,136 (31.1)
Positional titles	
Nurse	880 (24.1)
Nurse practitioner	1,550 (42.4)
Nurse-in-charge	1,121 (30.7)
Vice Professor	92 (2.5)
Chief nurse	11 (0.3)
Labor relationship	
Permanent nurse	994 (27.2)
Contract nurse	2,339 (64.0)
Others	321 (8.8)
Job burnout	
High emotional exhaustion (≥27)	2,148 (58.8)
High depersonalization (≥10)	697 (19.1)
Low personal accomplishment (≤33)	258 (7.1)

### Levels of occupational stress and job burnout

As presented in Table 2, the mean total occupational stress score was 64.19 ± 21.20. The average item score was 1.83 ± 0.61, corresponding to a mild-to-moderate stress level based on the scale’s criteria. Among the specific dimensions, “nursing profession and work” (2.18 ± 0.70) and “workload and time allocation” (2.13 ± 0.83) yielded the highest mean scores, whereas “nursing management and interpersonal relationships” was the lowest (1.52 ± 0.63).

**Table 2:** Characteristics of occupational stress and job burnout among nurses in Beijing (*N* = 3,654).

Variables	Items	Mean ± SD	Total score
Occupational stress	35	1.83 ± 0.61	64.19 ± 21.20
Nursing profession and work	7	2.18 ± 0.70	15.24 ± 4.93
Workload and time allocation	5	2.13 ± 0.83	10.65 ± 4.14
Work environment and resources	3	1.64 ± 0.75	4.93 ± 2.26
Patient nursing care	11	1.79 ± 0.68	19.68 ± 7.43
Nursing management and interpersonal relationships	9	1.52 ± 0.63	13.70 ± 5.63
Job burnout			
Emotional exhaustion	9	2.37 ± 0.65	21.33 ± 5.87
Depersonalization	5	2.01 ± 0.64	10.06 ± 3.20
Personal accomplishment	8	2.89 ± 0.53	23.13 ± 4.23

Regarding job burnout, the mean scores were 21.33 ± 5.87 for EE, 10.06 ± 3.20 for DP and 23.13 ± 4.23 for PA. Based on the specific clinical cut-off values adopted for this study (EE ≥ 27, DP ≥ 10, PA ≤ 33), the prevalence of burnout dimensions was substantial: 2,148 participants (58.8%) exhibited high EE; 697 participants (19.1%) exhibited high DP; and 258 participants (7.1%) reported low PA.

### Correlations between occupational stress and job burnout

Pearson correlation analysis (Table 3) revealed statistically significant correlations between all five dimensions of occupational stress and the three dimensions of job burnout (*p* < 0.01). Specifically, all stress dimensions showed moderate positive correlations with EE (*r* range: 0.436–0.542) and DP (*r* range: 0.345–0.489). Notably, stress related to “patient nursing care” showed the strongest correlation with EE (*r* = 0.542), while stress from “nursing management and interpersonal relationships” was most strongly correlated with DP (*r* = 0.489). Conversely, all occupational stress dimensions were weakly and negatively correlated with PA (*r* = −0.150 to −0.122).

**Table 3:** Pearson’s correlations of job burnout dimensions, and occupational stress scale (*N* = 3,654).

Variables	Nursing profession and work	Workload and time allocation	Work environment and resources	Patient nursing care	Nursing management and interpersonal relationships
Emotional exhaustion	0.493^**^	0.524^**^	0.436^**^	0.542^**^	0.526^**^
Depersonalization	0.349^**^	0.345^**^	0.381^**^	0.462^**^	0.489^**^
Personal accomplishment	−0.150^**^	−0.145^**^	−0.122^**^	−0.150^**^	−0.137^**^

*P < 0.05, **P < 0.01, ***P < 0.001.

### Multivariate analysis of factors associated with job burnout dimensions

The results of multivariate binary logistic regression analyses revealed distinct differential factors for the three burnout dimensions (Table 4). High EE was independently associated with all five occupational stress dimensions. The strongest associations were observed for “nursing management and interpersonal relationships” stress [Odds Ratio (OR) = 3.738, 95% CI: 2.294–6.089, *p* < 0.001] and “workload and time allocation” stress (OR = 2.801, 95% CI: 2.205–3.559, *p* < 0.001). High DP, conversely, was specifically linked to stressors arising from “patient nursing care” (OR = 2.401, 95% CI: 1.564–3.688, *p* < 0.001), “nursing management and interpersonal relationships” (OR = 2.036, 95% CI: 1.139–3.639, *p* = 0.016), and “work environment and resources” (OR = 1.486, 95% CI: 1.075–2.054, *p* = 0.017). For diminished PA, the analysis revealed that “nursing management and interpersonal relationships” stress was a significant risk factor (OR = 3.415, 95% CI: 1.772–6.583, *p* < 0.001). Notably, “workload and time allocation” showed a negative association with the outcome (OR = 0.556, 95% CI: 0.347–0.890, *p* = 0.014), indicating that higher workload stress was associated with lower odds of reporting reduced personal accomplishment in this cohort.

**Table 4:** Factors for job burnout by multivariate binary logistic regression of nurses in Beijing (*N* = 3,654).

Variables	Emotional exhaustion					Depersonalization					Personal accomplishment				
	*B*	SE	Wald	OR (95% CI)	*P*	*B*	SE	Wald	OR (95% CI)	*P*	*B*	SE	Wald	OR (95% CI)	*P*
Nursing profession and work	0.488	0.135	13.029	1.629 (1.250–2.124)	0.000	0.192	0.126	2.304	1.211 (0.946–1.551)	0.129	−0.196	0.247	0.629	0.822 (0.506–1.335)	0.428
Workload and time allocation	1.030	0.122	71.201	2.801 (2.205–3.559)	0.000	0.211	0.110	3.685	1.236 (0.996–1.533)	0.055	−0.588	0.240	5.977	0.556 (0.347–0.890)	0.014
Work environment and resources	0.339	0.163	4.304	1.403 (1.019–1.932)	0.038	0.396	0.165	5.735	1.486 (1.075–2.054)	0.017	0.238	0.282	0.715	1.269 (0.730–2.205)	0.398
Patient nursing care	0.934	0.186	25.139	2.545 (1.767–3.667)	0.000	0.876	0.219	16.018	2.401 (1.564–3.688)	0.000	0.356	0.324	1.208	1.428 (0.757–2.695)	0.272
Nursing management and interpersonal relationships	1.319	0.249	28.038	3.738 (2.294–6.089)	0.000	0.711	0.296	5.752	2.036 (1.139–3.639)	0.016	1.228	0.335	13.454	3.415 (1.772–6.583)	0.000

To verify the robustness of these findings and address potential information loss due to dichotomization, sensitivity analyses were performed using multiple linear regression on continuous burnout scores (see Supplementary Table S1). The results largely corroborated the primary logistic regression analysis for emotional exhaustion and depersonalization. Specifically, “nursing management and interpersonal relationships” and “workload and time allocation” remained the strongest factors associated with emotional exhaustion scores (*p* < 0.001). Similarly, the distinct associations for depersonalization were confirmed in the linear models, where management and patient care stressors were significant, but workload was not. However, regarding personal accomplishment, the results revealed a nuance: while the logistic model (focusing on the clinical threshold of low accomplishment) suggested a protective role for workload, the linear model showed a weak negative association (*β* = −0.058, *p* < 0.05), suggesting that the relationship between workload and accomplishment may vary depending on whether one examines the clinical risk threshold or the full score spectrum.

## Discussion

This study provides a systematic assessment of occupational stress and job burnout among nurses, offering an in-depth examination of the distinct association linking specific occupational stressors to individual burnout components. The key findings reveal a notable disconnection between perceived stress intensity and burnout severity, alongside clear differential associations whereby specific stress dimensions uniquely associated with distinct burnout components. These insights advance the understanding of the complex mechanisms underlying occupational health risks in nursing and offer critical empirical support for designing targeted interventions.

The surveyed nurses primarily comprised young, highly educated females employed on a contract basis, with bachelor’s degree attainment and monthly income exceeding 8,000 RMB, substantially higher than national averages (3). This profile reflects Beijing’s status as a first-tier city yet is accompanied by a high rate of contract-based employment, underscoring the professional instability within this demographic.

Nurses reported overall mild-to-moderate occupational stress levels, which were lower than those documented during the peak of the COVID-19 pandemic and in several earlier studies (16, 17). This reduction may be attributed to decreased emergency-induced overload and more stable policy support during the transition to regularized pandemic management. In stark contrast, job burnout remained severe, with particularly elevated scores in depersonalization and reduced personal accomplishment. Although emotional exhaustion scores showed some improvement from the extreme crisis period (18), the prevalence of high burnout (56.5% for EE, 39.8% for DP) remains alarming. This pattern of “moderate perceived stress alongside pronounced core burnout symptoms” suggests a shift in underlying mechanisms: while emotional exhaustion may dominate as an acute response during public health emergencies, chronic and structural stressors, such as limited career progression, diminished professional value, and complex workplace relationships, may more insidiously erode nurses’ professional identity and psychological efficacy in routine practice, manifesting as heightened depersonalization and persistently low personal accomplishment (19, 20).

The primary theoretical contribution of this study lies in its combined analytical approach, which moves beyond treating stress and burnout as monolithic constructs. Correlation analysis confirmed broad positive associations between all occupational stress dimensions and both emotional exhaustion and depersonalization, aligning with prior research (21, 22). However, multivariate regression analysis elucidated the specificity underlying these associations. Consistent with conservation of resources theory, emotional exhaustion was independently predicted by all five stress dimensions, suggesting that any stressor demanding psychological resources contributes to depletion (23, 24). The strongest associations were with stress from “workload and time allocation” and “nursing management and interpersonal relationships.” This indicates that emotional resource depletion is influenced not only by inherent clinical demands but more potently by inefficient time management, unreasonable task allocation, and, critically, a toxic organizational climate and interpersonal conflicts (25). Depersonalization was specifically associated with stress from “patient nursing care,” “work environment and resources,” and “nursing management and interpersonal relationships.” This pattern differs from studies emphasizing workload alone and supports the conceptualization of depersonalization as a defensive psychological detachment, particularly triggered by stressors that evoke helplessness and frustration, such as direct patient-related challenges, inadequate resource support, and poor organizational relationships (26, 27).

The most distinctive finding concerns diminished personal accomplishment. After controlling for other factors, only “nursing management and interpersonal relationships” stress and “workload and time allocation” stress were significantly associated with personal accomplishment. Specifically, we observed a divergent pattern: While stress from “nursing management and interpersonal relationships” was strongly associated with an increased risk of low personal accomplishment, stress from “workload and time allocation” showed a significant negative association, indicating a reduced likelihood of low personal accomplishment as workload stress increases. This finding, which may initially seem counterintuitive given the overall positive correlation between stress and burnout, suggests a nuanced relationship. It aligns with the challenge-hindrance stressor framework (28), positing that certain demands (like high workload) can be perceived as “challenges” that offer opportunities for growth, mastery, and a sense of achievement, thereby potentially preventing nurses from slipping into severe professional detachment. Conversely, “hindrance stressors” like interpersonal conflict and toxic management offer no such rewards and strictly undermine efficacy. It is important to note the nuance revealed by our sensitivity analysis (Supplementary Table S1). While the logistic model highlighted this protective effect against clinical burnout, the linear regression on continuous scores indicated a weak negative association. This suggests a complex threshold effect: high workload may act as a “challenge” that keeps nurses engaged enough to avoid the lowest levels of accomplishment (the clinical cutoff), yet the sheer volume of work still exerts a slight burden on their overall psychological reward system. However, given that the linear model explained a very low proportion of variance (*R*^2^ = 0.127) compared to the robust findings in the logistic model, the protective role of workload against severe burnout remains the primary clinical insight. Future research should explore the specific threshold at which “challenge” workload tips into “hindrance” overload.

### Limitations

This study has several limitations that should be considered. First, the cross-sectional design precludes causal inference. Although significant associations were identified, the directionality of relationships between occupational stress and burnout dimensions warrants verification through longitudinal research. Second, while the stratified sampling strategy covered diverse hospital types in Beijing with a high response rate (93.7%), the recruitment from selected institutions rather than a city-wide random sample may limit generalizability to rural or different cultural contexts. Third, methodological considerations regarding variable handling exist. We dichotomized continuous burnout scores to align with clinical management needs. While this approach can reduce statistical power, our large sample size and consistent results from sensitivity analyses using continuous scores (Supplementary Table S1) support the robustness of our core findings, particularly regarding emotional exhaustion and depersonalization, while revealing valuable nuances in the mechanisms of personal accomplishment. Finally, reliance on self-reported measures may introduce social desirability bias, despite strict anonymity. Future studies could benefit from integrating objective physiological stress indicators to validate self-reported data.

## Conclusion

The distinctiveness of this study lies in its revelation of the complex mechanisms through which occupational health risks transition from generalized stress perception to specific burnout components. Stress related to “nursing management and interpersonal relationships” consistently emerged as a core factor across all three burnout dimensions. This suggests that the primary bottleneck contributing to the deterioration of occupational health may have shifted from an absolute shortage of external resources to internal organizational “soft environment” factors, such as management efficacy, leadership quality, team atmosphere, and institutional fairness. Consequently, fostering supportive, equitable organizational cultures and harmonious interpersonal environments represents a “high-yield” strategy for alleviating nurse burnout and restoring professional value.

## Data Availability

The raw data supporting the conclusions of this article will be made available by the authors, without undue reservation.

## Footnotes

1
https://www.wjx.cn/
