# Supplementary material for: Differential associations of occupational stress dimensions with job burnout subscales among nurses
Source: Front Public Health. 2026 Mar 25;14:1774820. doi: 10.3389/fpubh.2026.1774820 (PMC13057389; doi:10.3389/fpubh.2026.1774820)
Supplement: Supplementary file 1 [file Table_1.DOCX]

**Supplementary Table S1. Sensitivity analysis: Multiple linear regression of occupational stress dimensions on continuous burnout scores (N = 3,654)**

| **Variables** | **Emotional Exhaustion (EE)** | **Depersonalization (DP)** | **Personal Accomplishment (PA)** |
| --- | --- | --- | --- |
|  | *β* (95% CI) | *β* (95% CI) | *β* (95% CI) |
| **Occupational Stress Dimensions** |  |  |  |
| Nursing Profession and Work | 0.104  (0.052 - 0.157) *** | 0.018  (-0.013 – 0.048) | -0.035  (-0.093 – 0.022) |
| Workload and Time Allocation | 0.209  (0.226 - 0.357) *** | -0.021  (-0.059 – 0.017) | -0.058  (-0.104 - -0.012) * |
| Work Environment and Resources | -0.050  (-0.152 - 0.052) | 0.055  (-0.005 – 0.114) | -0.001  (-0.091 – 0.022) |
| Patient Nursing Care | 0.153  (0.115 - 0.190) *** | 0.083  (0.061 – 0.105) *** | -0.023  (-0.063 – 0.018) |
| Management and Interpersonal | 0.217  (0.171 - 0.263) *** | 0.178  (0.151 – 0.205) *** | -0.034  (-0.067 - -0.001) * |
| **Covariates** |  |  |  |
| Age, Gender, Title, etc. | Adjusted | Adjusted | Adjusted |
| **Model Fit (****R^2^)** | 0.355 | 0.255 | 0.127 |

β = Standardized regression coefficient. Analyses were adjusted for age, gender, marital status, education, income, professional title, and employment type. * P < 0.05, ** P < 0.01, ***P < 0.001.
